# CRISPR, APGAR and Cancer Screening: Public Engagement, Controversy and Misaligned Values

**DOI:** 10.1111/bioe.70158

**Published:** 2026-07-23

**Authors:** Stephen John

**Affiliations:** ^1^ Department of History and Philosophy of Science University of Cambridge Cambridge UK

**Keywords:** cancer, deliberative democracy, ethics of screening, expertise, public engagement

## Abstract

There are many calls for public engagement in decision‐making around controversial technologies, but far fewer calls for public engagement in deliberation around less controversial interventions. We think the public should be involved in discussing CRISPR, but not in discussing APGAR. Drawing on the case of debates over cancer screening, this paper argues we have things the wrong way around. Given the complex entanglement of fact and value, it is very hard to have high‐quality public deliberation over novel technologies. By contrast, it is easier to have deliberation over more mundane technologies. There is, then, good reason to think we should engage in more public deliberation over mundane technologies. The results of this deliberation can then be used in more high‐profile cases.

CRISPR technologies for gene editing promise novel therapeutics for disease; however, sceptics are wary of ‘playing God’: how should CRISPR technologies be regulated? [1] APGAR scores provide a rough‐and‐ready assessment of newborn health, but some researchers object that they have low predictive value; should we use APGAR scores in routine neonatal care? [2] In discussions of the first sort of question—concerning novel technologies, controversial among the general public—many influential authors and institutions argue for extensive public engagement [3]. By contrast, few demand public engagement in discussions of mundane medical interventions, outside the public eye. In this paper, I argue that we have things the wrong way around. Rather, the complex entanglement of fact and value in a third case, routine cancer screening, implies a surprising conclusion: that we should have *more* public engagement in decision‐making about mundane medical interventions, and *less* in controversial cases.

In Section 1, I will sketch an argument *for* requiring public engagement in decision‐making over controversial cases but not in uncontroversial cases, based on what I call the ‘Democratic Model of expertise’. Ultimately, in Section 2, I reject this argument, because it rests on two dubious assumptions: that the sociological fact of controversy tracks ethical complexity, and that we can neatly distinguish between ethical and epistemic inputs into policymaking. In Sections 3 and 4, drawing on the systemic turn in deliberative democratic theory, I develop my own positive proposals, which take seriously the entanglement of epistemic and ethical expertise to argue for deliberation in—but only in—less controversial cases.

## 1 The Democratic Model of Expertise and the Appeal of Public Engagement

This section sketches an argument for the popular view that public engagement is more important when there is public controversy than when there is not. This argument has two parts: first, an argument for involving the public in decision‐making *at all*; second, an argument for *limiting* engagement to ‘controversial’ cases. Note that, I understand ‘public engagement’ to involve giving the public genuine input into, or genuine control over, policy decisions. Plausibly, such engagement might generate lots of goods; for example, it might make people better citizens by educating them about others' perspectives, or it might encourage technology adoption by making people feel involved. However, these goods might be secured *via* a sham process where we *pretend* citizens' views will guide policy. Therefore, I develop an argument for why *actual* engagement is valuable, in ways in which, say, a simulacrum of engagement would not be.^1^

Most policy questions about the introduction or use of medical technologies rest on highly technical scientific questions. One option is to leave these decisions to technical, scientific experts. Indeed, this is what we do in cases of ‘mandated science’ [4]. However, we do not leave all decisions to the scientists; furthermore, even when scientists have policymaking power, their mandate is typically constrained. To frame our discussion, then, it is helpful to sketch a broader account of the proper role of scientific experts in policy.

Most contemporary discussions of scientific expertise in policymaking stem, ultimately, from the seminal work of Max Weber [5, 6, 7]. Simplifying somewhat, Weber makes three key claims:

a. Policymaking (indeed, all decision‐making) requires two sorts of inputs: factual claims (i.e., claims about what is or could be the case) and evaluative claims (i.e., claims about which ends are valuable, and the constraints on pursuing those ends).
b. There can be experts on factual matters; that is, people who are better‐placed than others to identify true factual claims.
c. There are no experts on evaluative matters.

I will simply assume a. and b. I also assume that b. implies that ‘epistemic experts’ ought to play an important role in policymaking; we should grant their claims about factual matters far more weight than non‐experts' claims. There are, of course, problems with identifying ‘true’ epistemic experts; for example, because expertise can be gained through experience, rather than formal learning. However, I place these issues aside.

Why, though, hold c.: the claim that there are no experts on the correct values to use in policy‐making? This is a complex question, but here is one way to think about the issue. As Weber stressed, policy‐decisions often involve trading‐off ethical values [6]. For example, there are weighty reasons, linked to the promotion of health or the value of choice, to adopt a liberal approach towards CRISPR. However, there are also weighty reasons, grounded in respect for nature or precautionary reasoning, to adopt a more conservative approach. While wise policymaking may sidestep some trade‐offs, ultimately, policymaking requires *balancing* different values. A core feature of modern societies is that there is reasonable disagreement over such trade‐offs [8]. Acknowledging and respecting this disagreement implies scepticism about the possibility of identifying experts on value trade‐offs.

Assuming there are no ‘evaluative experts’, how should we identify the ‘correct’ values for policymaking? I assume that, ultimately, this question depends on an actual or ideal democratic procedure, which reflects the values of all affected parties. So, on what I call the *democratic model of expertise*, policymaking rests on factual inputs, which (where possible) ought to be provided by epistemic experts, and evaluative inputs, which, ultimately, ought to reflect the public's values, as settled through some actual or ideal democratic process. On this picture, scientitfic experts play an important, but limited, role in policymaking: they provide the factual inputs, but *only* the factual inputs. As Sections 2, 3, 4 explore, the idea that scientific expertise *can* be limited in this way is controversial. Still, the *democratic model of expertise* does seem assumed in much practice, where we routinely distinguish the scientific and ethical considerations in policymaking.

This very general model of the division of policy labour does not necessarily imply any particular claim about public engagement. Indeed, it is compatible with giving epistemic experts a high degree of ‘mandated authority’ where there is broad democratic agreement [4]. For example, if there is broad democratic agreement on the value of ensuring safe pharmaceuticals, we may leave pharmaceutical regulation to epistemic experts. Greater public engagement in regulatory decisions would be, at best, a waste of time, and even risky, as it might replace expert knowledge with uneducated hunches, or allow for cynical ‘gaming’ [9]. In other contexts, there are excellent reasons to think that the evaluative inputs to policy should be provided by elected politicians, rather than through actual public deliberation or engagement.

However, as in my CRISPR example, some areas of policymaking involve complex trade‐offs between competing values. In such cases, we cannot reasonably assume which trade‐offs would emerge through public deliberation, and we might be suspicious that electoral politics will capture the democratic will. In this context, some form of public engagement seems helpful as a guide for identifying the evaluative inputs into policy. While there is no guarantee that a mini‐public's deliberations will perfectly mimic a full democratic process, it seems preferable to alternatives [10].

On the *democratic model of expertise*, the key role for public engagement in policy‐making about medical technologies is to help us identify the correct trade‐offs between different values in ethically complex cases. When, though, are cases ethically complex? A plausible thought is that actual public controversy is what I call the *controversy‐tracks‐complexity principle*; that is, that greater ethical complexity is correlated with greater public controversy. For example, we might take the fact that CRISPR technologies *are* controversial as evidence of their ethical complexity. By contrast, we might take the fact that APGAR scores are not controversial as a reason to leave these decisions to epistemic experts. In the latter case, we might take a lack of public controversy as evidence that the broadly evaluative issues are clear; involving the public in deliberation would be a waste of resources, and might threaten the epistemic quality of deliberation.

I have outlined a way of justifying the widespread notion that public engagement is necessary in making decisions about controversial, but not uncontroversial, cases. One response would be to question the underlying denial of evaluative expertise [11]. In this paper, however, I will assume that there are no ultimate ethical experts. Rather, the next section challenges the *controversy‐tracks‐complexity principle*. I also start to question a fundamental premise of the *democratic model*: that we can ensure that scientific experts provide ‘epistemic inputs’ to policy without also implicitly shaping the evaluative inputs.

## 2 Screening: Controversy and Complexity

Routine mass screening of asymptomatic individuals for early disease has a been key tool of public health since the 1920s [12]. Furthermore, screening is very popular among the public; a recent survey showed that three‐in‐four adults in the UK wanted screening tests for cancer to be more widely available [13]. In this sort of non‐controversial case, decisions are often left to arms‐length bodies. This is true of screening; in the UK, decisions are largely delegated to the UK's National Screening Committee. Although there are calls for greater public engagement in screening decisions, typically, these calls are more concerned with establishing barriers to uptake, rather than providing insights to underlying value trade‐offs. However, I now argue that we should not confuse the sociological fact that programmes are controversial with the normatively relevant question of whether they are ethically complex. Rather, at least some ‘non‐controversial’ interventions, such as screening, *are* ethically complex.

Before going on, note a complexity: even if screening is non‐controversial among the public, many screening programmes are controversial *within the medical community* [14, 15]. Many medical researchers have worried about the evidence base for claims of efficacy, arguing that cancer screening may do ‘more harm than good’. In the second part of this section, I will argue that these epistemic controversies cannot be separated from the ethical problems I outline. These comments provide reasons to be cautious about the possibility that ‘epistemic experts’ might overstep the proper bounds of their expertise.

The basic underlying logic behind mass screening is simple: in general, the earlier that cancerous growths are detected, then the easier it is to treat them; hence, mass screening for early‐stage cancer should reduce population‐level cancer‐related morbidity and mortality. Note, however, that, *even if* cancer screening does save *some* lives, and, so, has important *benefits*, it also has *costs* [16]. Broadly, these fall into two categories: health‐related costs and psychosocial costs. Within the first category, we can identify two sub‐categories: first, iatrogenic harms associated with cancer screening modalities (such as exposure to radiation); second, costs arising from ‘overdiagnosis’ and ‘overtreatment’. Even characterising the second sub‐category is contentious, but, broadly, it refers to cases where individuals are treated for early‐stage cancers which would not have gone on to cause mortality and morbidity in the absence of intervention (e.g., women diagnosed with a ductal carcinoma in situ who have a preventative mastectomy, where the carcinoma would not have developed into a tumour) [17]. Within the category of psychosocial costs, we might include the ‘costs’ of anxiety associated with ‘false positive’ results, but, also, the more general phenomenon of medicalisation or pathologisation of healthy individuals.

Plausibly, a necessary condition for introducing a cancer screening programme is that the expected ‘benefits’ outweigh the expected ‘costs’. However, identifying ‘the costs’ of cancer screening is epistemically costly: calculating rates of ‘overdiagnosis’ is no simple matter. I leave these epistemic issues aside for now, to note it is also ethically complex; first, we need to decide whether, say, ‘anxiety’ or ‘medicalisation’ really *is* a cost *at all*; second, if so, we need to find some way in which to commensurate this cost against putative health‐related benefits. *Even if* we sidestep these problems and restrict attention to ‘medical’ costs, we face problems. While it seems clear that victims of overtreatment *are* harmed by participation in cancer screening programmes, the relevant harms are *invisible*; because we can never say of some individual that *her* treatment was unnecessary, a victim of overtreatment will never be aware that she suffered unnecessary harm; indeed, subjectively, she may be extremely glad that she received treatment.

Call the demand that interventions do more good than harm *Beneficence*. Thinking about screening requires us to make difficult decisions about how to specify this consideration. They also drag in other ethical concerns. While all medical interventions have opportunity costs, a key feature of screening programmes is that they *directly* harm some patients. Normally, we hold; however, physicians may not impose uncompensated harm on their patients, even if this imposition produces benefits for other patients. Physicians involved in screening seem to violate their duty of *Non‐Maleficence* [16]. Hence, any judgment of the permissibility of screening must involve balancing two competing ethical demands. One possible response to this problem is to shift our ethical attention away from the *outcomes* of screening to focus, instead, on its effects on individuals' chances of harm and benefit; we could say that, even if cancer screening will *harm* some *ex post*, it is a justifiable intervention because (or, at least, when) it is in the *ex ante* interests of each affected individual. However, the question of whether we ought to focus ethical attention on *ex ante* or *ex post* assessment is hotly contested, and a plausible position holds that we must balance *both* perspectives [18].

Furthermore, just as cancer screening raises questions about the balance between *Beneficence* and *Non‐Maleficence*, so, too, it raises questions about other important ethical values. For example, one obvious response to concerns that cancer screening harms individuals is that these harms are permissible as long as participants gave fully informed consent to participation. Unfortunately, securing informed consent in the context of screening is complex, because doing so requires patients to understand complex statistical information. Furthermore, there the fact that screening encounters are typically initiated by medical professionals, in the form of an invitation, itself affects informed consent. It is an open question whether and how screening programmes respect *Autonomy* [19]. Finally, given differential uptake of screening across social classes, screening programmes may increase health inequalities. As such, even if screening programmes clearly do more good than harm, meeting the demands of *Beneficence*, they may be in tension with (some accounts of) *Justice*.

Given the difficulties of establishing the effects of early intervention on ‘all‐cause mortality’ and measuring the incidence of overdiagnosis, assessing the effects of cancer screening is epistemically complex [15]. I have argued, however, that assessing cancer screening policies is also ethically complex. Even worse, these ethical and epistemic complexities are interrelated. Consider, for example, the question of *which* costs and benefits to count; there may be no ‘neutral’ way of presenting the ‘outcomes’ of screening which does not (implicitly) presuppose which *sorts* of outcomes are relevant to ethical deliberation [14]. Furthermore, questions about the epistemic uncertainties that plague epidemiological research into screening may be embroiled with different ethical perspectives. It is notable that proponents of clinical epidemiology, steeped in a tradition which stresses *primum non nocere*, tend to be far more sceptical of screening than traditional epidemiologists, steeped in a more consequentialist mindset [19].

## 3 Public Engagement, Screening and the Problem of Misalignments

In Section 1, I sketched an account of why public engagement may seem important in controversial, high‐profile cases: because the fact of public controversy signals that those cases are ethically complex, and public engagement is a good guide to the democratically correct trade‐offs between ethical values in complex cases. Section 2 showed that the *controversy‐tracks‐complexity* principle is too simple: although uncontroversial among the general public, cancer screening is ethically complex. One example does not show that there is no correlation between controversy and complexity. Furthermore, nothing I have said shows that CRISPR cases are not *even more* complex. I return to these issues below. In this section, I will continue to focus on screening. Here is one obvious conclusion from Sections 1 and 2: we ought to ensure greater public engagement in screening policy. In the previous section, however, I also suggested that, in practice, it is hard to disentangle ethical and epistemic aspects of cancer screening debates. I will now show how this epistemic‐ethical entanglement poses challenges for effective public engagement.

Consider two, highly simplified, models of the relationship between epistemic experts and public representatives in debates over cancer screening, which I call ‘values first’ and ‘facts first’. On a ‘values first’ model, roughly, the public representatives ought to engage in some deliberation over which values ought to be pursued, and how they ought to be balanced, before handing these results to the scientific experts to identify effective policies. Unfortunately, this approach seems implusible. First, there are very general problems with identifying what counts as ‘the’ public's values in the first place [20]. Furthermore, even if we could reliably identify ‘public values’ in some abstract way, interpreting and applying general principles to specific cases is not, itself, an ethically straightforward task. It is one thing to be told that screening programmes ought not create unnecessary anxiety, or ought to balance *Beneficence* and *Non‐Maleficence*, but quite another to know what this means in deciding between, say, biennial or triennial breast cancer screening. The ethical devil lies in the practical detail. A ‘values first’ model leaves too much freedom for epistemic experts to shape policy. This leeway violates the *democratic model of expertise*, because it means that the scientists, rather than the public, shape the evaluative inputs to policy.

No‐one holds to a strict ‘values first’ approach to public engagement. Rather, more typically, public engagement is structured such that experts present a neutral outline of some of the key issues, before public deliberation starts [21]. However, this ‘facts first’ model also faces challenges. There are different ways of understanding these challenges. To set out the basic problem, I will rely on the concept of ‘misalignments’ [7]. To explain, we can think of the process of researching and reporting a topic as involving a series of epistemically underdetermined choices, that is, where more than one option is compatible with basic epistemic norms. So, for example, given limited resources, researchers may have to choose between studying overdiagnosis rates *or* the number of lives ‘saved’ by screening; either option is compatible with basic epistemic norms. Similarly, in preparing a brief overview, researchers may have to choose between reporting statistics on anxiety or on uptake in ethnic minority groups; again, purely epistemic concerns do not dictate this choice. As a matter of fact, these ‘choices’ may be heavily constrained (say, by funding, or local norms). However, we can reconstruct them ‘as if’ they reflected certain evaluative stances; for example, a sense that overdiagnosis rates or rates of anxiety are *more ethically weighty* than lives saved or ethnic minority uptake.

Unlike deliberate fraud or bias, the fact that scientists' work is ‘as if’ value‐laden is not necessarily a problem. Rather, it is an unavoidable aspect of *all* scientific research and reporting. However, this pheonemon creates a problem when scientists inform the public about some topic, with the aim of, ultimately, eliciting the public's considered evaluative views. Imagine that a scientist simply fails to report any concerns about equity in screening (it is ‘as if’ she thinks equity is unimportant). However, were they aware of equity concerns, members of a mini‐public would view this as an important consideration in screening policy. In this context, there is a ‘misalignment’ between the values implicit in the scientist's research and the democratically correct values (i.e., the values the public would endorse after full deliberation). However, when the scientist fails to report equity concerns, actual deliberators are unlikely to voice these concerns; the misalignment renders actual public engagement a bad guide to the correct democratic values for policymaking.

The risk of misalignments is unavoidable. Scientists cannot research *all* of the topics or report *all* of the findings. Even if they could, the resulting ‘data dump’ would be as likely to undermine as to promote informed deliberation [22]. We might, of course, hope that scientific experts could align their research agendas and reporting practices with what the public do, or ought, to care about [23]. Again, however, this is no easy task. Consider the fact that many people seem to feel much more fear about cancer, ‘the big C’, than other diseases [24]. Many epidemiological experts, however, view this sort of ‘onco‐exceptionalism’ as an irrational bias. In this case, it is not clear whether the epistemic experts *ought* to treat cancer as ‘special’ in research and communication. Doing so seems to reflect what many in the public *do* value (and, hence, might ensure that resulting deliberation will adequately capture citizens' values); on the other hand, ‘aligning’ to these concerns also risks undermining proper deliberation by simply reinforcing prejudices. A choice must be made, but it is unclear which option best ensures that public deliberation is well‐informed. Misalignments are hard to manage, but threaten to prejudice debate in favour of certain evaluative perspectives, sidelining or even silencing other evaluative perspectives, hence undermining the rationale for public engagement.

The possibility of misalignments does not show that we should never seek public engagement in decisions around screening. After all, the alternative— leave it to the experts—is also problematic. However, recognising this possibility has an important implication. Many authors have proposed models for ensuring that the public is well‐informed by epistemic experts; for example, James Fishkin has agued that it is important that debate moderators do not reveal their own positions and that briefing documents should be written by committees representing a wide range of viewpoints [21]; Ronald Pielke has argued that scientific advisors should seek to be ‘honest brokers’, who provide a range of alternative perspectives [25]. Some authors have suggested more radical approaches, where, rather than present a well‐worked scientific consensus, publics should be made aware of scientific disagreement. For example, in recent work on technical decision‐making, Zeynep Pamuk has suggested that we might structure some decisions along a legal model, where public representatives must choose between two (or more) competing experts, making very different policy recommendations [26].

I suggest that, if we are concerned by the possibility of misalignments, we should prefer these more radical proposals, because they provide our best hope for revealing the ‘as if’ value judgments inherent in scientists' claims, therefore revealing misalignments. We should not have a ‘facts‐first’ model, but create a situation where the assumptions behind ‘the facts’ are open to ‘value‐based’ criticism.

## 4 Beyond Screening

Section 1 reconstructed an argument for the view that we should seek greater public engagement in deciding ‘controversial’ cases (but not in ‘uncontroversial cases’). Section 2 argued that the controversial/uncontroversial distinction does not perfectly track the ethically complex/ethically simple distinction: therefore, the argument also implies a need for public engagement in *some* uncontroversial cases. Section 3 developed an account of how to structure such engagement, alert to the possibility of ‘misalignments’. However, the fact that some uncontroversial cases are complex doesn't show that controversial cases are ethically simple; maybe, for example, CRISPR cases are *even more complex* than screening cases. Therefore, my arguments so far seem to imply a broadening of public engagement, to include *both* controversial cases and at least some uncontroversial cases. However, I will now argue that the issues discussed in Section 3 pose a significant challenge to public engagement in high‐profile cases; the factors which make public engagement difficult in mundane cases pose even more significant challenges in publicly controversial cases, and the mechanisms which might mitigate *some* of these dangers in simple cases are likely to be *even less* effective in high‐profile cases. The second part of this section proposes a response to this ‘controversy problem’, drawing on the ‘systemic turn’ in deliberative democratic theory.

We can summarise the general lesson of the previous section as pointing to a tension between two conditions which must be met for public engagement to be a valuable input to policymaking. The first is:

a. Public deliberation must be well‐informed
  Simply talking about ‘values’ in an abstract way may not provide action‐guiding recommendations. Rather, public engagement must grapple with the details of proposed interventions and policies. The second condition is:
b. Public deliberation must be autonomous
  Where by ‘autonomous’ I mean to capture the notion that deliberation should be free from external influences, such as threats or coercion. In our context, deliberation can fail to be autonomous when there are ‘surreptious value influences’ on deliberation.

Each of (a) and (b) is independently demanding. The previous section argued that, given possible ‘misalignments’, it is extremely difficult to meet both conditions *simultaneously*. In trying to meet (a) we run into problems with (b), and vice versa. So, for example, we need the public to have a good grasp of the details of different cancer screening modalities if they are to make well‐informed decisions about cancer‐screening policies. However, at the same time, the more we demand scientists tell the public, the more likely there will be some ‘misalignments’, ensuring surreptitious value influence. I suggested that adopting a more contestatory model for scientific input to deliberation might help mitigate some of these tensions. However, these approaches are not guaranteed to succeed in revealing *all* misalignments. Ensuring high‐quality public engagement is difficult when we are dealing with relatively mundane, familiar interventions like cancer screening.

I now suggest that meeting these conditions is likely to be even harder in cases involving controversial technologies. Often, controversy surrounds novel technologies. Clearly, when these technologies *are* novel, it is hard to meet condition (a). We cannot merely provide publics with a high‐level account of what some intervention involves in order to ascertain their value trade‐offs, because it may be unclear how to apply those vague claims to concrete decisions; for example, a vague evaluative claim that ‘CRISPR is good, as long as we are reasonably sure germline interventions are safe’ leaves open a series of value‐laden questions about what counts as ‘reasonably sure’ or ‘safe’. Unfortunately, in high‐profile cases we often lack the sort of evidence that allows us to make predictions about things like long‐term effects. Of course, there is a similar problem in cases like cancer screening, where there is scientific controversy over the effects of screening. However, our epistemic situation is likely to be *even worse* when we consider novel technologies, where we are not only uncertain how to interpret data, but also lack data to interpret in the first place. These concerns may be slightly different in cases of controversial technologies which are not novel; say, for example, the use of the MMR vaccine. However, in those sorts of cases, educating citizens is hard for a different reason; when older technologies *are* controversial, the public may have been exposed to a huge amount of putative information, much of which is misleading.^2^

Second, consider condition (b). One form of surreptitious value influence occurs when scientists' testimony implicitly favours certain values. However, clearly this is not the only form of surreptitious value influence. Rather, we also know that deliberation may be influenced by interested actors, such as profit‐seeking corporations. This occurs in cases of routine drug approvals [9]. Given that new technologies are often tied into hopes of massive financial reward, these risks are likely to be even greater when discussing controversial cases. Note that this is not to say that (discussions of) mundane technologies are *not* influenced by corporate power, but, rather, that, simply in virtue of a long history, we are typically better‐placed to identify and mitigate this influence.

These two problems are related; ensuring that public deliberation is well‐informed may be even harder when most of those asked to do the ‘informing’ have strong interests to ‘push’ for—or against—some technology. This is always a risk in any form of public deliberation. However, the risk is higher in cases of technologies where the boundaries between science, industry and political agendas are highly blurred. This is not to impugn individual scientists' motivations; rather, it is to point to the fact that the entire research agenda may have been shaped to generate certain sorts of information—say, broadly ‘favouring’ some intervention—in ways which may not track what public representatives would, given their values, want to know [27]. It is difficult enough to meet (a) and (b) simultaneously in *uncontroversial* cases, such as cancer screening; meeting them in *controversial* cases, where the stakes are much higher is far harder again.

I suggested possible responses to similar problems in the case of screening; we should encourage contestation in order to highlight the ethical values and perspectives which may implicitly shape epistemic experts' work. However, these mechanisms risk backfiring with more controversial interventions. The core aim of public engagement is, ultimately, to come to some kind of agreement on how to trade‐off different evaluative considerations. Such deliberation is particularly difficult when interventions are already controversial, because a pro‐ or anti‐ attitude may, already, be related to individuals' political identity; this is true both of novel technologies and of established technologies [28]. Adding contestation *between* scientists to public engagement risks exacerbating these problems; by presenting decisions in an adversarial way, we increase the possibility that participants will be ill‐informed—relying on short‐cut heuristics about what ‘people like me’ support—and non‐autonomous—swayed by interested parties, rather than the force of deliberation. While contestation may sometimes be effective for recognising the complex evaluative issues at play in ‘uncontroversial’ cases, it is likely to exacerbate the problems with debating ‘controversial’ cases. We face the ‘controversy problem’: public engagement is most difficult at precisely the point where it seems most important.

I do not have a simple solution to this problem. However, I suggest that the ‘systemic turn’ in deliberative democracy may suggest a partial reply [29, 30]. Since the 1990s, many theorists have argued that political authority and legitimacy must be grounded in democratic deliberation. On the face of it, this approach seems to suggest a radical opening‐up of political debate. However, opening‐up is often ill‐advised; justifying policies in public does not necessarily meet the high standards of ‘public reason’ but can devolve into ‘plebiscitary reason’, where politicians appeal to ungrounded fears or engage in other forms of rhetoric [31]. At least sometimes, keeping political deliberation ‘behind closed doors’ seems the best way to ensure it is high‐quality. One response to these tensions has been to suggest that we ought to take a more ‘systemic’ perspective on public justification. Rather than assume that a commitment to deliberative democracy implies that *all* parts of the political system *ought* to be open to public debate and deliberation, instead, we should ask how different parts of a system, which might include large areas ‘closed’ from actual public scrutiny, work together. As an example, consider the concept of ‘mandated authority’ from Section 1. We might hold that, ultimately, political justification must be grounded in deliberation, but isolate regulatory agencies from actual deliberation on the grounds that this best promotes democratic goals.

In a helpful contribution, Alfred Moore has suggested a general model for thinking about technical experts in democracy, ‘critical elitism’, according to which we should delegate large amounts of political authority to technical experts, but only when those experts' claims are *open to* possible democratic debate and criticism [32]. In turn, Moore suggests that a degree of public scepticism in scientific expertise may play an important political role, in ensuring that experts are forced to respond to public concerns and criticisms. In a similar vein, Greg Lusk has argued that concerns about how ‘value‐laden’ climate science can be used in democratic contexts may be resolved if we think in systemic terms [33].

I suggest that a systemic focus may also help us respond to the controversial problem. Given the *democratic model of expertise* when making decisions about controversial new interventions, any evaluative inputs should reflect what the public do or would value. However, plausibly, actual public engagement is more likely to distort reasoning as to improve it. There are good reasons to keep these decisions ‘behind closed doors’. How, though, can we ensure that the ‘value inputs’ to those deliberations reflect the ‘correct’ democratic values, the values which would be endorsed through a proper democratic procedure?

One solution to this problem is to have robust forms of public engagement around other, non‐controversial, interventions. Having these debates clarifies the sorts of trade‐offs which the public is willing to make, the ways in which they are likely to respond to various forms of uncertainty, and creates a vivid awareness among technical experts of the limits of a technocratic world‐view. These all help improve ‘closed’ deliberation in controversial cases. Broadly, there are two (compatible) models we could use for this sort of ‘transfer’. One model is to hold that ‘behind closed doors’ deliberation on controversial topics ought to be attended by a small number of laypeople who have been involved in deliberation in other, non‐controversial cases. The function of these laypeople would not be to act as representatives of the public as a whole, but to act as a kind of rapporteur, pointing out which sorts of reasons were offered and the agreements made in the more mundane case. A second possibility would be to delegate this work to trained bio‐ethicists or social scientists, who might be better placed to interpret the arguments which arose in deliberation in mundane cases, and to reason how these sorts of concerns apply to the novel case. Either way, the key feature of the system would be that deliberation in mundane cases is used as an ‘input’ into deliberation on the controversial cases, without *actual* public engagement. This approach follows Moore's model—where expert trustworthiness is guaranteed by experts' responsiveness in some cases—rather than Pamuk's approach, which holds *all* decisions ought to be open to contestation.

Given the *democratic model of expertise*, in a very wide range of cases public engagement might, in principle, be helpful. Unfortunately, public engagement always runs a risk of misalignments. The best response to this problem is to engage in contestatory forms of public engagement. These work badly in controversial cases. Therefore, we have two reasons for engaging in public engagement in the uncontroversial cases: first, to help us make better policy in those cases; second, to help ensure we make better decisions in the controversial cases. Just as deliberative democrats need not hold that we must open *all* decisions up to democratic debate, so, too, we can recognise the important reasons for public engagement without resolving controversial cases via public engagement.

Before concluding, it is worth noting two potential worries about my arguments. First, I have claimed that *actual* public engagement over controversial matters is likely to be sub‐optimal. However, you might worry that experts are *also* under pressure, which may skew their judgment, such that deliberation over controversial technologies to occur behind closed doors will also be sub‐optimal; for example, the risk of corporate capture of expert decision‐making may be greater when deliberation is closed from public engagement, or experts might ignore some of the genuine concerns underlying allegedly ‘irrational’ public fears. I admit that there is no guarantee that expert deliberation, over controversial cases will not be skewed. However, the hope we might shield expert deliberation from broader media and corporate pressures is more plausible than the hope of shielding public deliberation from those pressures, if only because there are well‐established systems for enculturating professional norms in experts which are lacking in the broader public. Furthermore, a lack of public engagement is not necessarily the same as complete opacity; there are many mechanisms short of *actual* engagement, such as forms of accountability, for holding officials to account [34].

A second worry is that my proposals may seem likely to cause public consternation, because they may seem elitist or patronising; one could caricature the arguments above as holding that the public are ‘too stupid’ or ‘too easily swayed’ to reason carefully about controversial technologies. Clearly, citizens might object to this characterisation, in turn threatening their willingness to recognise the legitimacy of expert decisions. Unsurprisingly, I dispute this characterisation: my proposals do not assume that epistemic experts are inherently wiser than non‐experts, but rest on the sociological observation that circumstances of controversy often make it difficult for non‐experts to engage in well‐informed, autonomous debate. However, even if my arguments are *not* patronising, they might be *perceived* as such. This is a genuine risk. Note, however, that many important policy decisions are made by small groups of ‘experts’, shielded from the public eye. For example, decisions about interest rates are often set by central banks [35]. These systems are not normally treated as paternalistic or insulting. The key trick may be to identify how such institutions maintain perceived legitimacy, and to use similar techniques to justify my proposed approach.

## 5 Conclusion

All decisions about introducing and regulating medical technologies and interventions involve both factual and evaluative claims. In turn, basic democratic norms imply that the relevant evaluative claims ought to be grounded ultimately in the public's values. This is true both of novel, highly controversial interventions, such as CRISPR gene‐editing techniques, and of highly uncontroversial cases, such as using APGAR scores on neonatal wards. When, then, should we use *actual* public engagement to guide decision‐making? Section 1 sketched an argument for a popular position, which we can summarise, crudely, as ‘CRISPR cases, but not APGAR cases’. Section 2 suggested, however, that some ‘uncontroversial’ cases are, in fact, far more complex than they seem. It seems that the answer might be ‘CRISPR cases and APGAR cases’. Section 3 explored some of the difficulties with proper public engagement in uncontroversial cases, leading to the unfamiliar position developed in Section 4, “APGAR cases, but not CRISPR”.

Is that too neat? Of course it is. First, there may be good reasons, grounded in concerns about perceived political legitimacy, to use public engagement in many high‐profile, controversial cases. Second, just because screening is more complex than it may seem, it does not follow that *all* ‘uncontroversial’ cases call for public deliberation. Indeed, plausibly, the use of APGAR scores may be something best left to ‘the experts’. Still, the arguments in this paper do establish more modest conclusions. First, cancer screening is an ethically complex area. It reminds us that we should be suspicious that public controversy is necessarily a guide to ethical complexity. Second, in debates over cancer screening, ethical and epistemic issues are intertwined in complex ways. This should make us think harder about how public engagement can be *both* informed and autonomous. Neither result shows that we *must* or *must not* engage in any specific form of public engagement in any specific case. They do, however, suggest that we should think of public engagement as part of more complex systems, rather than as a response to—but only to—controversy.

More generally, my arguments have implications for bioethicists. My claims are unlikely to change practice. Still, my conclusions have an important implication for how we think about the practice of bioethics. For a variety of reasons, we often end up focusing our attention on ‘high‐profile’ controversial cases, preferring to debate CRISPR rather than cancer screening. There may be some good arguments for this practice. However, in focusing on the controversial, we risk making it seem as if *only* the controversial cases are difficult. Just as I have proposed that the scientific experts should spend more time thinking about dull topics, the same may be true of the ethicists.^3^

## Data Availability

The authors have nothing to report.

## Endnotes

1 These comments raise difficult questions about the relationship between ‘perceived’ and ‘real’ legitimacy. For current purposes, we can place these issues to one side; my concern is not to deny the importance of ‘perceived legitimacy’ but the more mundane observation that there is a difference between *believing* something is happening and that thing happening. Thanks to an anonymous reviewer for pushing on these issues.

2 Thanks to an anonymous reviewer for pushing me to distinguish more clearly between cases of novel and familiar controversial technologies.

3 This paper received very helpful feedback from a commentator, Drew Schroeder, from the audience at an online event organised by Mark Sheehan and Nir Eyal, and from two reviewers for this journal. Thanks to all of them.
